# Analysis of Covid-19 data using discrete Marshall–Olkinin Length Biased Exponential: Bayesian and frequentist approach

**DOI:** 10.1038/s41598-023-39183-6

**Published:** 2023-07-28

**Authors:** Hassan M. Aljohani, Muhammad Ahsan-ul-Haq, Javeria Zafar, Ehab M. Almetwally, Abdulaziz S. Alghamdi, Eslam Hussam, Abdisalam Hassan Muse

**Affiliations:** 1 Department of Mathematics and Statistics, College of Science, Taif University, P.O. Box 11099, 21944 Taif, Saudi Arabia; 2 College of Statistical and Actuarial Sciences, University of the Punjab, Lahore, Pakistan; 3 Faculty of Business Administration, Delta University of Science and Technology, Gamasa, 11152 Egypt; 4 Scientific Association for Applied Studies and Research (SAASR), Al Manzalah, Egypt; 5 College of Science and Arts, King Abdulaziz University, P. O. Box 344, 21911 Rabigh, Saudi Arabia; 6 Department of Mathematics, Faculty of Science, Helwan University, Helwan, Egypt; 7 Faculty of Science and Humanities, School of Postgraduate Studies and Research (SPGSR), Amoud University, Borama, 25263 Somalia

**Keywords:** Pure mathematics, Statistics

## Abstract

The paper presents a novel statistical approach for analyzing the daily coronavirus case and fatality statistics. The survival discretization method was used to generate a two-parameter discrete distribution. The resulting distribution is referred to as the "Discrete Marshall–Olkin Length Biased Exponential (DMOLBE) distribution". Because of the varied forms of its probability mass and failure rate functions, the DMOLBE distribution is adaptable. We calculated the mean and variance, skewness, kurtosis, dispersion index, hazard and survival functions, and second failure rate function for the suggested distribution. The DI index demonstrates that the proposed model can represent both over-dispersed and under-dispersed data sets. We estimated the parameters of the DMOLBE distribution. The behavior of ML estimates is checked via a comprehensive simulation study. The behavior of Bayesian estimates is checked by generating 10,000 iterations of Markov chain Monte Carlo techniques, plotting the trace, and checking the proposed distribution. From simulation studies, it was observed that the bias and mean square error decreased with an increase in sample size. To show the importance and flexibility of DMOLBE distribution using two data sets about deaths due to coronavirus in China and Pakistan are analyzed. The DMOLBE distribution provides a better fit than some important discrete models namely the discrete Burr-XII, discrete Bilal, discrete Burr-Hatke, discrete Rayleigh distribution, and Poisson distributions. We conclude that the new proposed distribution works well in analyzing these data sets. The data sets used in the paper was collected from 2020 year.

## Introduction

A new pandemic appeared at the beginning of 2019 till March 2020 called the COVID-19 (Feroze^1^). Because of the lockout, COVID-19 has the greatest impact on human life and the economy. Pakistan is the 12th most impacted country in the world as a result of COVID-19 (Khan et al.^2^).

Mathematical models for the analysis of infectious disease transmission are currently omnipresent. Such models’ play a significant role in assisting with quantifying conceivable irresistible aliment control. Several models are available for infectious disease concerning compartmental models, beginning from the classical SIR model to more complex proposals (Ndaïrou et al.^3^).

To model the count data sets there are several classical probability distributions such as Binomial, Poisson, Geometric, and Negative Binomial distributions but these models do not provide a better fit for the over-dispersed nature of data sets. Hence one way to deal with such data sets is to discretize the continuous model dealing with the specific behavior to have a better fit. Discretization has attained much attention in the last few decades due to its applicability and better fitting for count data analysis.

In past several discrete distributions have been introduced and studied, such as discrete Weibull distribution discrete beta exponential distribution by Nekoukhou et al.^4^, two-parameter discrete Lindley distribution by Hussain et al.^5^, Discrete Marshall–Olkin inverse Toppe–Leone with application to COVID-19 data has been obtained by Almetwally et al.^6^. discrete weighted exponential distribution by Rasekhi et al.^7^ exponentiated discrete Lindley distribution by El-Morshedy et al.^6^, discrete Burr Hutke distribution by El-Morshedy et al.^8^, discrete Marshall–Olkinin Weibull distribution by Opone et al.^9^), see Almetwally et al.^10^, discrete Marshall–Olkinin alpha power inverse Lomax distribution by Almetwally et al.^11^, discrete inverted Topp–Leone distribution by Eldeeb et al.^12^, discrete Ramos–Louzada distribution by Eldeeb et al.^13^, discrete type-II half logistic exponential distribution Ahsan-ul-Haq et al.^14^, discrete power-Ailamujia distribution by Alghamdi et al.^15^, Poisson XLindley distribution Ahsan-ul-Haq et al.^16^, Poisson moment exponential distribution Ahsan-ul-Haq^17^ and discrete moment exponential distribution by Afify et al.^18^.

Discrete extended odd Weibull exponential with the application of COVID-19 Mortality Numbers in the Kingdom of Saudi Arabia and Latvia has been introduced by Nagy et al.^19^. The pmf of the new model for a mixture representation of a geometric model has been obtained by El-Morshedy et al.^20^.

All these discrete probability models are introduced using the survival discretization approach. Let a random variable X be associated with a continuous probability distribution having survival function $S\left(x\right)$. The probability mass function (pmf) of a discrete random variable based on discretization is

1 $$P\left(X = x\right)= S\,\left(x\right)- S\,\left(x+1\right), \,\,\,\,\,\,\,\,\,x=\mathrm{0,1},\mathrm{2,3},\dots$$

The primary purpose of this study is to introduce a new flexible probability distribution for modelling across over-dispersed data sets. The mathematical properties of the new distribution, such as its simple closed-form expressions for the pmf, cdf, moments, and other characteristics, are obtained. The maximum likelihood approach is used to estimate the model parameters. To suggest a new alternative approach to model over dispersed data sets, the DMOLBE distribution applied to the number of deaths due to Covid-19 data sets. Consequently, the DMOLBE model's primary goals are:

- The fact that this distribution provides the several hazard rate forms, such as declining, growing, or increasing-constant, sets it apart from many other one- or two-parameter discrete distributions. Because of these hazard rates, the suggested model can be used to model a variety of data sets.
- It provides a variety of PMF shapes suitable for modelling symmetric, positively skewed, or negatively skewed data that may not be successfully modelled by other competitor models.
- The introduction of a number of statistical and reliability traits, such as moments, probability functions, reliability indices, hazard functions, reverse hazard rate, second rate of Failure, etc.
- In comparison to other discrete distribution models in the literature, analysis results from two practical applications revealed that the DMOLBE distribution matches the supplied data sets satisfactorily;
- In the presence of gathered data, maximum likelihood and Bayesian estimation methods are taken into consideration to estimate the specified parameters.
- The effectiveness of the acquired estimators is assessed using lengthy Monte Carlo simulations and a variety of accuracy metrics, including mean squared errors and absolute biases. It would seem plausible to suggest that approaches for parameter estimation are adequate and efficient.

The study is divided into the following sections: “Methodology” is based on the mathematical characteristics and derivation of the discrete Marshall–Olkinin Length Biased Exponential distribution. “Parameter estimation” presents maximum likelihood estimation via an extensive simulation study. “Bayesian estimation” discusses the results for all models. Finally, in “Results and discussion”, we bring the research to a close.

## Methodology

In this section, we introduced a new discrete distribution, derived its statistical properties, estimate the model parameters using the maximum likelihood approach.

### The DMOLBE distribution and its properties

Let X be a random variable connected with the Ahsan-ul-Haq et al.^21^ presented Marshall–Olkinin Length Biased Exponential distribution. The MOLBE distribution's probability density function is:

2 $$f\,\left(x\right)=\frac{\gamma \frac{x}{{\beta }^{2}}{e}^{-\left(\frac{x}{\beta }\right)}}{{\left[1-\left(1-\gamma \right)\left(1+\frac{x}{\beta }\right){e}^{-\left(\frac{x}{\beta }\right)}\right]}^{2}}, x>0,\gamma >0,\beta >0.$$

The associated survival function is

3 $$S\,\left(x\right)=\frac{\gamma \left(1+\frac{x}{\beta }\right){e}^{-\left(\frac{x}{\beta }\right)}}{1-\left(1-\gamma \right)\left(1+\frac{x}{\beta }\right){e}^{-\left(\frac{x}{\beta }\right)}}.$$

The DMOLBE distribution obtained using Eqs. (1) and (3), the pmf of the DMOLBE distribution is given

4 $$P\left(x\right)=\frac{\gamma \left[\left(1+\frac{x}{\beta }\right){e}^{-\left(\frac{x}{\beta }\right)}- \left(1+\frac{x+1}{\beta }\right){e}^{-\left(\frac{x+1}{\beta }\right)}\right] }{\left\{1-\left(1-\gamma \right)\left(1+\frac{x}{\beta }\right){e}^{-\left(\frac{x}{\beta }\right)}\right\} \left\{1-\left(1-\gamma \right)\left(1+\frac{x+1}{\beta }\right){e}^{-\left(\frac{x+1}{\beta }\right)}\right\}}.$$

The cdf of DMOLBED is as follows

5 $$F\left(x\right)=\frac{ \left\{1-\left(1+\frac{x+1}{\beta }\right){e}^{-\left(\frac{x+1}{\beta }\right)} \right\} }{ \left\{1-\left(1-\gamma \right)\left(1+\frac{x+1}{\beta }\right){e}^{-\left(\frac{x+1}{\beta }\right)}\right\}}, x>0,\gamma >0,\beta >0.$$

where $\gamma$ is shape and $\beta$ is scale parameter.

**Figure 1:**
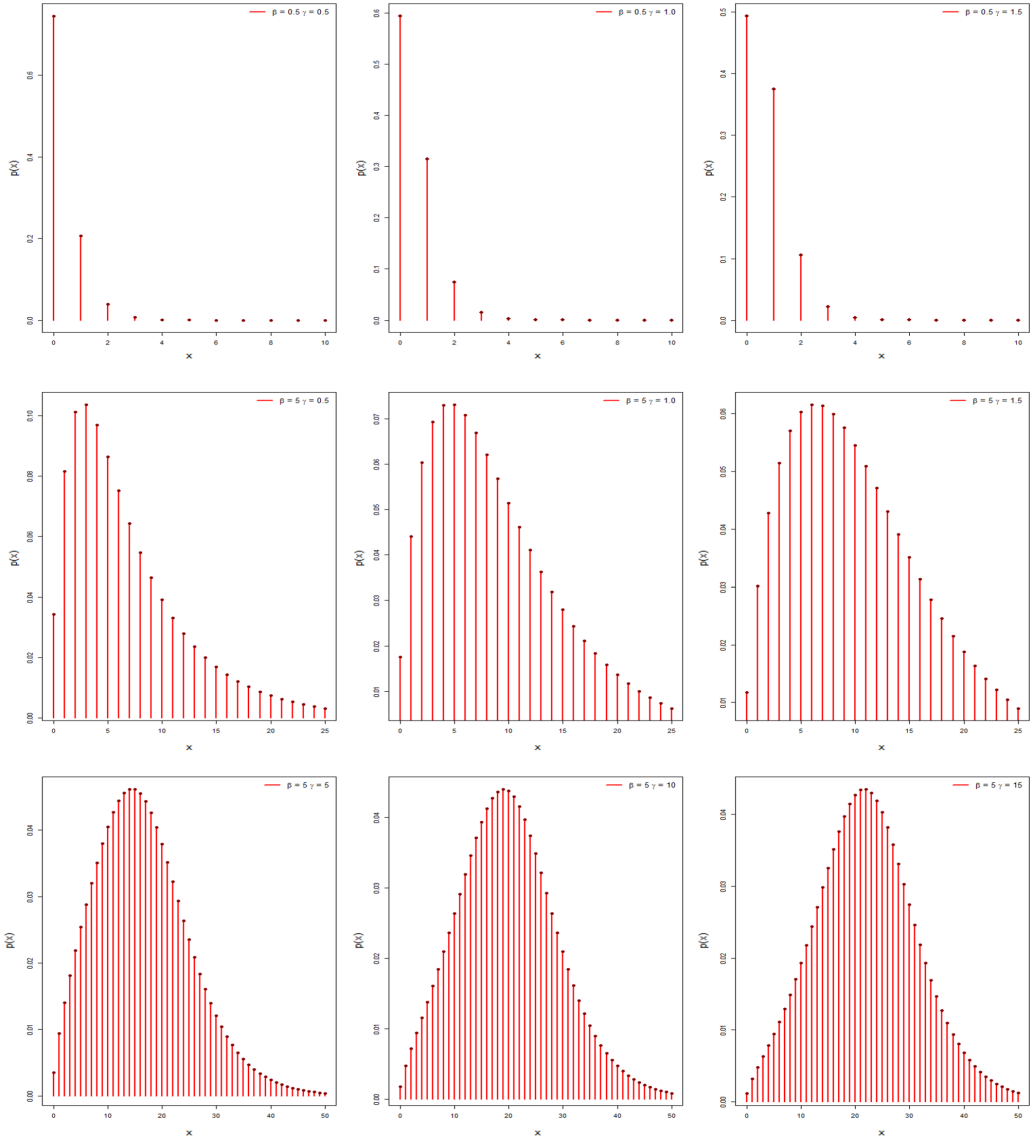
The pmf plots of DMOLBE distribution.

Figure 1 depicts the behavior of the probability mass function of the DMOLBE distribution, which varies with parameter values. The DMOLBE distribution is clearly declining, positively skewed, and symmetric, as seen above. It demonstrates the suggested distribution's versatility in dealing with data of varying behaviour.

### Survival and hazard function

The survival function of DMOLBED is as follows

6 $$S\left(x\right)=\frac{ \gamma \left(1+\frac{x+1}{\beta }\right){e}^{-\left(\frac{x+1}{\beta }\right)} }{ \left\{1-\left(1-\gamma \right)\left(1+\frac{x+1}{\beta }\right){e}^{-\left(\frac{x+1}{\beta }\right)}\right\}}.$$

The hazard function (hrf) of DMOLBE is given as follows

7 $$h\left(x\right)=\frac{\left[\left(1+\frac{x}{\beta }\right){e}^{-\left(\frac{x}{\beta }\right)}- \left(1+\frac{x+1}{\beta }\right){e}^{-\left(\frac{x+1}{\beta }\right)}\right] }{\left(1+\frac{x+1}{\beta }\right){e}^{-\left(\frac{x+1}{\beta }\right)} \left\{1-\left(1-\gamma \right)\left(1+\frac{x}{\beta }\right){e}^{-\left(\frac{x}{\beta }\right)}\right\} }.$$

**Figure 2:**
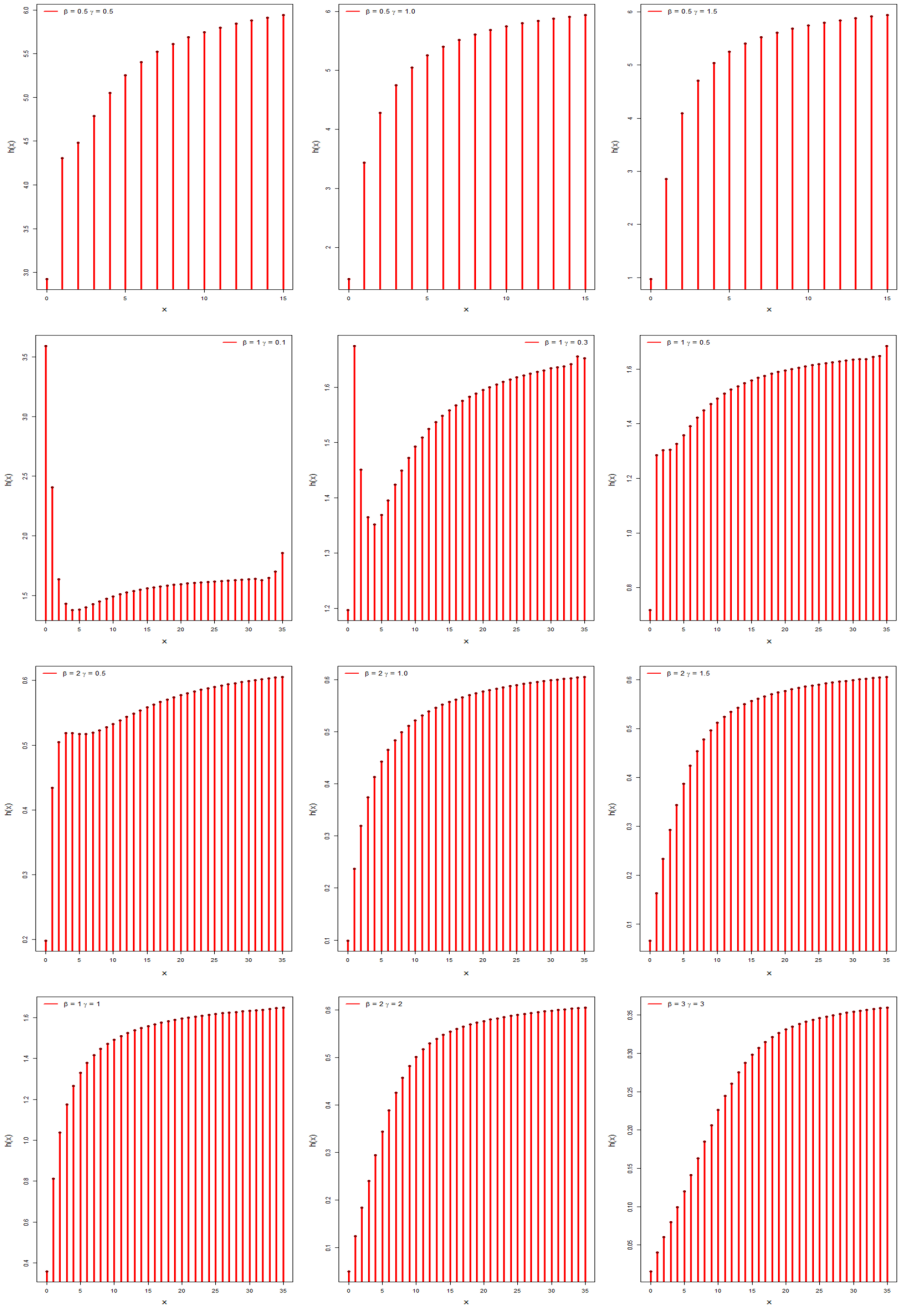
The discrete hrf plots of DMOLBE distribution.

Figure 2 shows the behavior of hazard function for different values of parameters which is increasing and decreasing which shows the flexibility of the model.

### The second rate of failure

The second rate of failure of DMOLBE is defined as

$$SRF=\mathrm{log}\left[\frac{G\left(x\right)}{G\left(x+1\right)}\right],$$

8 $$SRF=\mathrm{log}\left[\frac{\left(\beta +x+1\right){e}^{\left(\frac{1}{\beta }\right)} \left\{1-\left(1-\gamma \right)\left(1+\frac{x+2}{\beta }\right){e}^{-\left(\frac{x+2}{\beta }\right)}\right\} }{ (\beta +x+2) \left\{1-\left(1-\gamma \right)\left(1+\frac{x+1}{\beta }\right){e}^{-\left(\frac{x+1}{\beta }\right)}\right\}}\right].$$

### Reverse hazard rate

The reverse Hazard rate of DMOLBE is defined as:

9 $${r}^{*}\left(x\right) = \frac{P\left(x\right)}{F\left(x\right)}=\frac{\gamma \left[\left\{\left(1+\frac{x}{\beta }\right){e}^{-\left(\frac{x}{\beta }\right)}\right\}- \left\{\left(1+\frac{x+1}{\beta }\right){e}^{-\left(\frac{x}{\beta }+1\right)}\right\}\right] }{\left\{1-\left(1-\gamma \right)\left(1+\frac{x}{\beta }\right){e}^{-\left(\frac{x}{\beta }\right)}\right\} \left\{1-\left(1+\frac{x+1}{\beta }\right){e}^{-\left(\frac{x+1}{\beta }\right)}\right\}}.$$

### Recurrence formula

10 $$\frac{P(x+1)}{P(x)}= \frac{\left[\left(1+\frac{x+1}{\beta }\right){e}^{-\left(\frac{x+1}{\beta }\right)}- \left(1+\frac{x+2}{\beta }\right){e}^{-\left(\frac{x+2}{\beta }\right)}\right] \left\{1-\left(1-\gamma \right)\left(1+\frac{x}{\beta }\right){e}^{-\left(\frac{x}{\beta }\right)}\right\}}{ \left\{1-\left(1-\gamma \right)\left(1+\frac{x+2}{\beta }\right){e}^{-\left(\frac{x+2}{\beta }\right)}\right\}\left[\left\{\left(1+\frac{x}{\beta }\right){e}^{-\left(\frac{x}{\beta }\right)}\right\}- \left\{\left(1+\frac{x+1}{\beta }\right){e}^{-\left(\frac{x+1}{\beta }\right)}\right\}\right]}.$$

### Probability generating function and moments

Let X be a discrete random variable, then the probability generating function of the DMOLBE distribution is given as follows:

11 $${G}_{x}\left(z\right)=\sum_{x=0}^{\infty }{Z}^{x}P\left(X\right)=1+\gamma \left(z-1\right)\sum_{x=1}^{\infty }{z}^{x-1} \frac{\left(1+\frac{x}{\beta }\right){e}^{-\left(\frac{x}{\beta }\right)}}{1-\left(1-\gamma \right)\left(1+\frac{x}{\beta }\right){e}^{-\left(\frac{x}{\beta }\right)}}.$$

Differentiating ${G}_{x}\left(Z\right)$ with respect to $Z$ and setting $Z=1$, we can obtain the factorial moments as

$${G}_{x}^{\prime}\left(1\right) = \gamma \sum_{x=1}^{\infty }\frac{\left(1+\frac{x}{\beta }\right){e}^{-\left(\frac{x}{\beta }\right)}}{1-\left(1-\gamma \right)\left(1+\frac{x}{\beta }\right){e}^{-\left(\frac{x}{\beta }\right)}},$$

$${G}_{x}^{{\prime}{\prime}}\left(1\right) =2\gamma \sum_{x=1}^{\infty }(x-1) \frac{\left(1+\frac{x}{\beta }\right){e}^{-\left(\frac{x}{\beta }\right)}}{1-\left(1-\gamma \right)\left(1+\frac{x}{\beta }\right){e}^{-\left(\frac{x}{\beta }\right)}},$$

$${G}_{x}^{{\prime}{\prime}{\prime}}\left(1\right) = 3 \gamma \sum_{x=1}^{\infty }\left(x-1\right)\left(x-2\right) \frac{\left(1+\frac{x}{\beta }\right){e}^{-\left(\frac{x}{\beta }\right)}}{1-\left(1-\gamma \right)\left(1+\frac{x}{\beta }\right){e}^{-\left(\frac{x}{\beta }\right)}},$$

$${G}_{x}^{{\prime}{\prime}{\prime}{\prime}}\left(1\right) = 4 \gamma \sum_{x=1}^{\infty }\left(x-1\right)\left(x-2\right)(x-3) \frac{\left(1+\frac{x}{\beta }\right){e}^{-\left(\frac{x}{\beta }\right)}}{1-\left(1-\gamma \right)\left(1+\frac{x}{\beta }\right){e}^{-\left(\frac{x}{\beta }\right)}},$$

The factorial moments can be used to compute moments about the origin.

$$\mu ={G}_{x}^{\prime}\left(1\right)=\gamma \sum_{x=1}^{\infty }\frac{\left(1+\frac{x}{\beta }\right){e}^{-\left(\frac{x}{\beta }\right)}}{1-\left(1-\gamma \right)\left(1+\frac{x}{\beta }\right){e}^{-\left(\frac{x}{\beta }\right)}},$$

$${\mu }_{2}^{\prime}= {G}_{x}^{{\prime}{\prime}}\left(1\right) + {G}_{x}^{\prime}\left(1\right),$$

$${\mu }_{2}^{\prime}=\gamma \sum_{x=1}^{\infty }(2x-1) \frac{\left(1+\frac{x}{\beta }\right){e}^{-\left(\frac{x}{\beta }\right)}}{1-\left(1-\gamma \right)\left(1+\frac{x}{\beta }\right){e}^{-\left(\frac{x}{\beta }\right)}},$$

$${\mu }_{3}^{\prime} = {G}_{x}^{{\prime}{\prime}{\prime}}\left(1\right)+ 3{G}_{x}^{{\prime}{\prime}}\left(1\right) + {G}_{x}^{\prime}\left(1\right),$$

$${\mu }_{3}^{\prime}=\gamma \sum_{x=1}^{\infty }\left(3{x}^{2}-3x+1\right) \frac{\left(1+\frac{x}{\beta }\right){e}^{-\left(\frac{x}{\beta }\right)}}{1-\left(1-\gamma \right)\left(1+\frac{x}{\beta }\right){e}^{-\left(\frac{x}{\beta }\right)}},$$

$${\mu }_{4}^{\prime}= {G}_{x}^{{\prime}{\prime}{\prime}{\prime}}\left(1\right)+ 6{G}_{x}^{{\prime}{\prime}{\prime}}\left(1\right) + 7{G}_{x}^{{\prime}{\prime}}\left(1\right) + {G}_{x}{\prime}\left(1\right),$$

$${\mu }_{4}^{\prime}=\gamma \sum_{x=1}^{\infty }\left(4{x}^{3}-6{x}^{2}+4x-1\right) \frac{\left(1+\frac{x}{\beta }\right){e}^{-\left(\frac{x}{\beta }\right)}}{1-\left(1-\gamma \right)\left(1+\frac{x}{\beta }\right){e}^{-\left(\frac{x}{\beta }\right)}},$$

Now variance is

$${\sigma }^{2}={\mu }_{2}^{\prime}-{\left({\mu }_{1}^{\prime}\right)}^{2},$$

$${\sigma }^{2}=\gamma \sum_{x=1}^{\infty }(2x-1) \frac{\left(1+\frac{x}{\beta }\right){e}^{-\left(\frac{x}{\beta }\right)}}{1-\left(1-\gamma \right)\left(1+\frac{x}{\beta }\right){e}^{-\left(\frac{x}{\beta }\right)}}- {\left(\gamma \sum_{x=1}^{\infty }\frac{\left(1+\frac{x}{\beta }\right){e}^{-\left(\frac{x}{\beta }\right)}}{1-\left(1-\gamma \right)\left(1+\frac{x}{\beta }\right){e}^{-\left(\frac{x}{\beta }\right)}}\right)}^{2},$$

and the coefficients of skewness (CS) and kurtosis (CK) may be computed as follows

$$CS=\frac{{\mu }_{3}^{\prime}-3{\mu }_{2}^{\prime}\upmu +2{\upmu }^{3}}{{\left({\sigma }^{2}\right)}^\frac{3}{2}},$$

$$CK=\frac{{\mu }_{4}^{\prime}{-4\mu }_{3}^{\prime}\upmu +6{\mu }_{2}^{\prime}{\upmu }^{2}-3{\upmu }^{4}}{{\left({\sigma }^{2}\right)}^{2}}.$$

**Figure 3:**
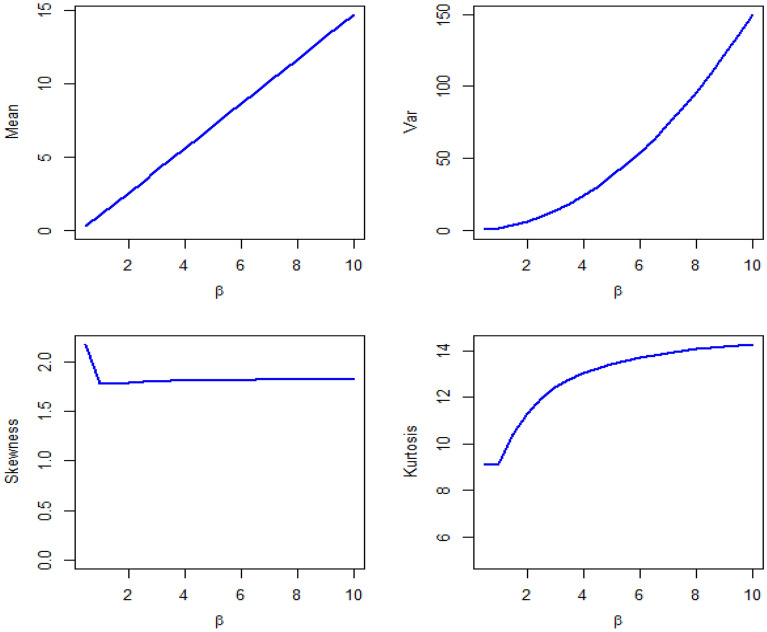
Plots of mean, variance, skewness and kurtosis of DMOLBED $\left(\gamma =0.5\right)$

**Figure 4:**
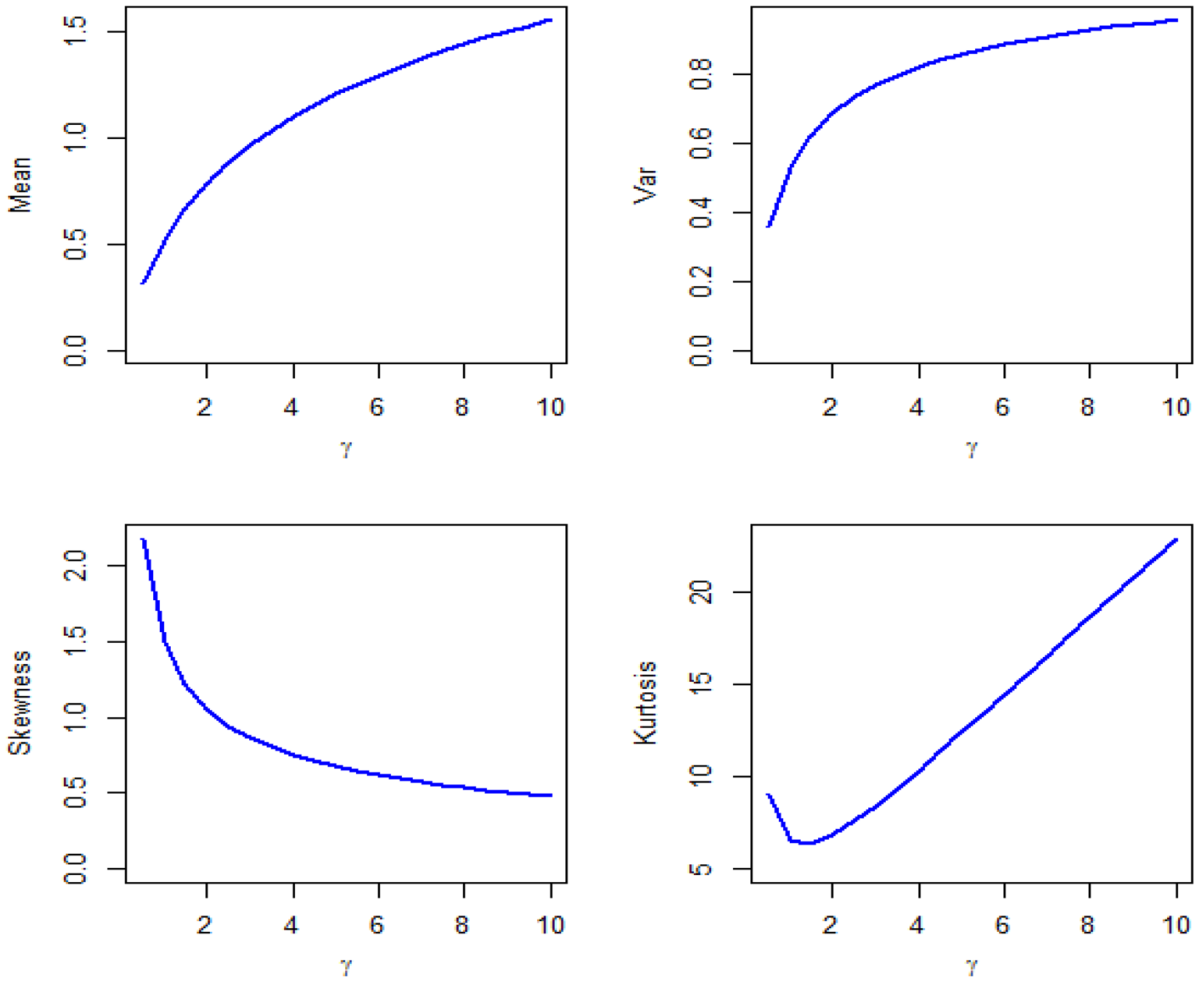
Plots of mean, variance, skewness and kurtosis of DMOLBED $\left(\beta =0.5\right)$

**Figure 5:**
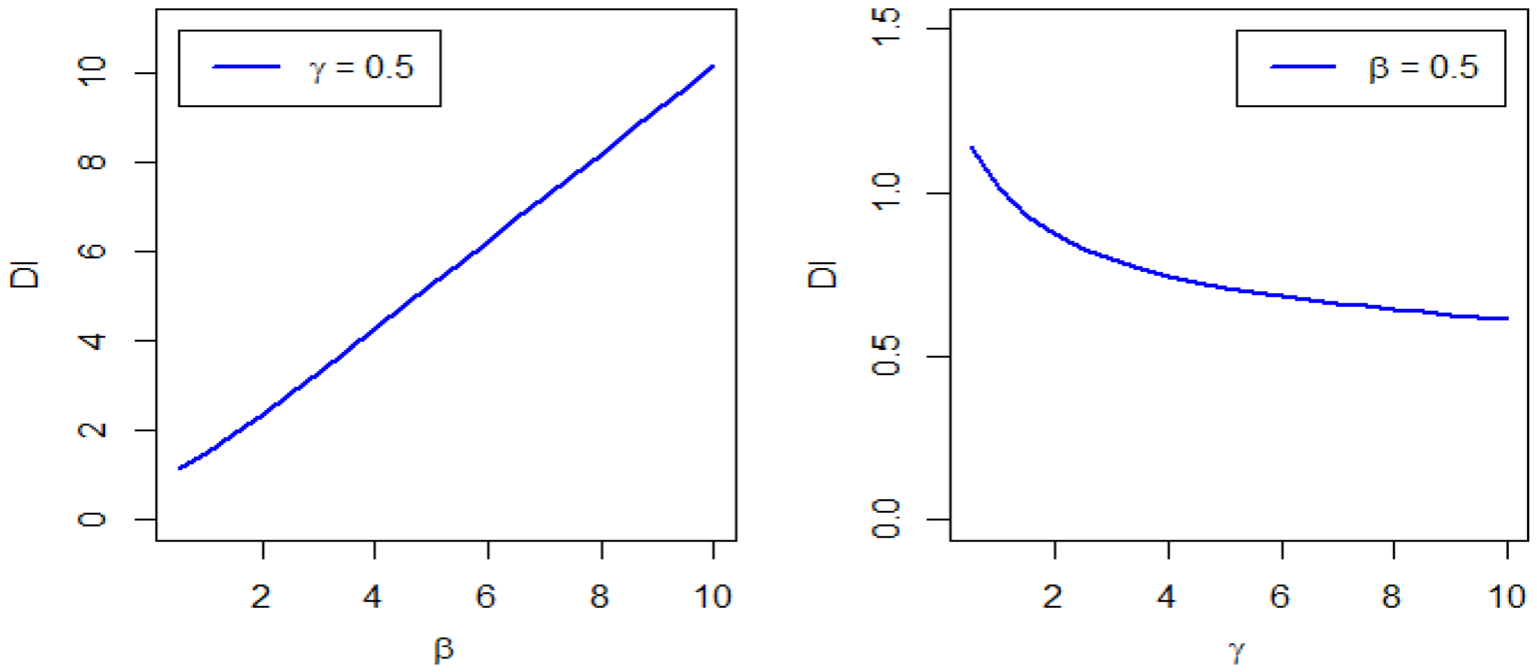
Plots of dispersion index of DMOLBED.

By Table 1 and Figs. 3, 4, and 5 show different measures of moment with different values of parameters.

**Table 1:** Different measures of moment with different values of parameters.

$\beta$	$\gamma$	$\mu$	${\mu }_{2}^{\prime}$	${\mu }_{2}$	${\mu }_{3}^{\prime}$	${\mu }_{4}^{\prime}$	$CS$	$CK$
0.4	0.4	0.46	0.54	0.3284	0.7	1.02	0.7942	2.6265
0.4	0.8	0.72	1	0.4816	1.68	3.4	0.7974	3.7328
0.4	1.4	0.88	1.24	0.4656	2.08	4.12	0.5330	3.5097
0.4	2	1	1.48	0.48	2.56	5.08	0.3608	3.1250
0.4	4	1.28	2.2	0.5616	4.4	9.88	0.3476	2.9354
0.8	0.4	1.06	1.98	0.8564	4.66	13.02	0.9408	3.8485
0.8	0.8	1.46	3.38	1.2484	9.98	34.82	1.0036	3.9361
0.8	1.4	1.82	4.94	1.6276	17.06	69.98	1.0329	4.1700
0.8	2	2.04	5.72	1.5584	19.68	78.92	0.8496	3.7884
0.8	4	2.54	8.5	2.0484	34.22	159.22	0.7586	3.7434
1.4	0.4	1.94	6.38	2.6164	28.94	162.14	1.5149	5.7179
1.4	0.8	2.56	10	3.4464	51.04	312.88	1.2182	4.5966
1.4	1.4	3.18	14.5	4.3876	83.22	561.46	1.0016	3.9426
1.4	2	3.58	17.54	4.7236	105.7	735.86	0.8850	3.5072
1.4	4	4.42	25.22	5.6836	169.42	1284.02	0.5686	3.0929
2	0.4	2.78	12.9	5.1716	82.7	646.98	1.5376	5.4719
2	0.8	3.68	20.68	7.1376	152.48	1351.24	1.2505	4.6498
2	1.4	4.46	28.3	8.4084	225.38	2109.34	0.9909	3.9480
2	2	5.08	35.64	9.8336	307.6	3081.48	0.8639	3.6358
2	4	6.4	52.48	11.52	508.48	5559.52	0.6433	3.0650
4	0.4	5.52	50.36	19.8896	635.4	9822.92	1.5539	5.5989
4	0.8	7.3	80.98	27.69	1189.78	21,106.42	1.3338	4.8751
4	1.4	9.08	117.4	34.9536	1912.16	36,477.64	1.0230	3.8559
4	2	10.26	143.86	38.5924	2494.98	50,398.9	0.9471	3.7760
4	4	12.72	208.32	46.5216	4045.92	89,269.92	0.6699	3.2863

The corresponding Dispersion Index (DI) is defined as

$$DI=\frac{Variance\,of\,DMOLBED}{Mean\,of\,DMOLBED}$$

The DI indicates whether a distribution is suitable to model over or under-dispersed data sets. If $DI>1$, the certain distribution is showing over-dispersed behavior. It is observed that the DMOLBE distribution shows over-dispersion when $\gamma =0.5$ and different values of parameter $\beta$. Conversely, the DMOLBE distribution shows under-dispersion when $\beta =0.5$ and different values of $\gamma .$

## Parameter estimation

Suppose $x=({x}_{1}, {x}_{2}, {x}_{3}, \dots , {x}_{n} )$ be a random sample of size n from DMOLBE distribution with probability mass function defined as

$$P\left(x\right)=\frac{\gamma \left[\left\{\left(1+\frac{x}{\beta }\right){e}^{-\left(\frac{x}{\beta }\right)}\right\}- \left\{\left(1+\frac{x+1}{\beta }\right){e}^{-\left(\frac{x+1}{\beta }\right)}\right\}\right] }{\left\{1-\left(1-\gamma \right)\left(1+\frac{x}{\beta }\right){e}^{-\left(\frac{x}{\beta }\right)}\right\} \left\{1-\left(1-\gamma \right)\left(1+\frac{x+1}{\beta }\right){e}^{-\left(\frac{x+1}{\beta }\right)}\right\}}.$$

Then the log-likelihood function is given by:

12 $$\mathrm{log}L=n\mathrm{log}\left(\upgamma \right)+\sum_{i=1}^{n}\mathrm{log}\left[\left\{\left(1+\frac{{x}_{i}}{\beta }\right){e}^{-\left(\frac{{x}_{i}}{\beta }\right)}\right\}- \left\{\left(1+\frac{{x}_{i}+1}{\beta }\right){e}^{-\left(\frac{{x}_{i}+1}{\beta }\right)}\right\}\right]-\sum_{i=1}^{n}\mathrm{log}\left[1-\left(1-\gamma \right)\left(1+\frac{{x}_{i}}{\beta }\right){e}^{-\left(\frac{{x}_{i}}{\beta }\right)}\right]-\sum_{i=1}^{n}\mathrm{log}\left[1-\left(1-\gamma \right)\left(1+\frac{{x}_{i}+1}{\beta }\right){e}^{-\left(\frac{{x}_{i}+1}{\beta }\right)}\right],$$

Now partially differentiate w.r.t γ and β, respectively.

13 $$\frac{\partial L}{\partial \gamma }=\frac{n}{\gamma }-\sum_{i=1}^{n}\frac{\left(1+\frac{{x}_{i}}{\beta }\right){e}^{-\left(\frac{{x}_{i}}{\beta }\right)}}{\left\{1-\left(1-\gamma \right)\left(1+\frac{{x}_{i}}{\beta }\right){e}^{-\left(\frac{{x}_{i}}{\beta }\right)}\right\}}-\sum_{i=1}^{n}\frac{\left(1+\frac{{x}_{i}+1}{\beta }\right){e}^{-\left(\frac{{x}_{i}+1}{\beta }\right)}}{\left\{1-\left(1-\gamma \right)\left(1+\frac{{x}_{i}+1}{\beta }\right){e}^{-\left(\frac{{x}_{i}+1}{\beta }\right)}\right\}},$$

14 $$\frac{\partial L}{\partial \beta }=\sum_{i=1}^{n}\frac{ \frac{{{x}_{i}}^{2}}{{\beta }^{2}}{e}^{-\left(\frac{{x}_{i}}{\beta }\right)}- \frac{{\left({x}_{i}+1\right)}^{2}}{{\beta }^{2}} {e}^{-\left(\frac{{x}_{i}+1}{\beta }\right)}}{\left(1+\frac{{x}_{i}}{\beta }\right){e}^{-\left(\frac{{x}_{i}}{\beta }\right)}- \left(1+\frac{{x}_{i}+1}{\beta }\right){e}^{-\left(\frac{{x}_{i}+1}{\beta }\right)} }+\sum_{i=1}^{n}\frac{\left(1-\gamma \right) \left(\frac{{{x}_{i}}^{2}}{{\beta }^{2}}\right){e}^{-\left(\frac{{x}_{i}}{\beta }\right)}}{1-\left(1-\gamma \right)\left(1+\frac{{x}_{i}}{\beta }\right){e}^{-\left(\frac{{x}_{i}}{\beta }\right)}}+\sum_{i=1}^{n}\frac{\left(1-\gamma \right)\frac{{\left({x}_{i}+1\right)}^{2}}{{\beta }^{2}}{e}^{-\left(\frac{{x}_{i}+1}{\beta }\right)}}{1-\left(1-\gamma \right)\left(1+\frac{{x}_{i}+1}{\beta }\right){e}^{-\left(\frac{{x}_{i}+1}{\beta }\right)}}.$$

Since it is difficult to find a closed-form solution for the set of nonlinear Eqs. (13, 14) with unknown gamma and beta values, the above-described nonlinear system may be numerically solved using an iterative method like Newton–Raphson by ‘maxLik’ package in R software.

## Bayesian estimation

Since random and parameter uncertainty are expressed by a prior joint distribution that was generated before the data was obtained on the failure, the Bayesian approach deals with parameters. The flexibility of the Bayesian technique to incorporate previous knowledge into research makes it particularly useful in the study of reliability, as the lack of data is one of the major problems with reliability analysis. The $\gamma$ and $\beta$ parameters of DMOLBED take prior gamma distributions, where $\gamma$ and $\beta$ are non-negative values. The α and b parameters as independent joint prior density functions can be expressed as follows:

$$\pi \left(\gamma ,\beta \right)\propto {\gamma }^{{a}_{1}-1}{e}^{-\gamma {b}_{1}}{\beta }^{{a}_{2}-1}{e}^{-\beta {b}_{2}}.$$

The estimates and their variances were equated with the inverse of the Fisher information matrix of alpha and beta to produce the ML estimator for $\gamma$ and $\beta$, which was contributed by Dey et al.^23^. This procedure was used to extract the hyper-parameters of the informative priors. The joint posterior density function of $\gamma$ and $\beta$ are derived from likelihood function of DMOLBED and joint prior density:

15 $$\pi \left(\gamma ,\beta |x\right)\propto {\gamma }^{{a}_{1}}{e}^{-\gamma {b}_{1}}{\beta }^{{a}_{2}-1}{e}^{-\beta {b}_{2}}\frac{\left\{\left(1+\frac{x}{\beta }\right){e}^{-\left(\frac{x}{\beta }\right)}\right\}- \left\{\left(1+\frac{x+1}{\beta }\right){e}^{-\left(\frac{x+1}{\beta }\right)}\right\} }{\left\{1-\left(1-\gamma \right)\left(1+\frac{x}{\beta }\right){e}^{-\left(\frac{x}{\beta }\right)}\right\} \left\{1-\left(1-\gamma \right)\left(1+\frac{x+1}{\beta }\right){e}^{-\left(\frac{x+1}{\beta }\right)}\right\}}.$$

Most Bayesian inference processes have been created using symmetric loss functions. The squared-error loss function is a popular symmetric loss function. The Bayes estimators of $\gamma$ and $\beta$, say $\widetilde{\gamma }$ and $\widetilde{\beta }$ based on squared error loss function is given by

$$\widetilde{\gamma }={\int }_{0}^{\infty }\gamma {\int }_{0}^{\infty }\pi \left(\gamma ,\beta |x\right) d\beta\,d\gamma , \widetilde{\gamma }={\int }_{0}^{\infty }\beta {\int }_{0}^{\infty }\pi \left(\gamma ,\beta |x\right) d\gamma\,d\beta .$$

See Almetwally et al.^22^ employed the MCMC technique to solve the above equations.

Two of the most prevalent MCMC methodologies are the Metropolis–Hastings (MH) and Gibbs sampling methods. We employ the MH inside the Gibbs sampling stages:

16 $$\pi \left(\gamma |\beta ,x\right)\propto {\gamma }^{{a}_{1}}{e}^{-\gamma {b}_{1}}\frac{\left\{\left(1+\frac{x}{\beta }\right){e}^{-\left(\frac{x}{\beta }\right)}\right\}- \left\{\left(1+\frac{x+1}{\beta }\right){e}^{-\left(\frac{x+1}{\beta }\right)}\right\} }{\left\{1-\left(1-\gamma \right)\left(1+\frac{x}{\beta }\right){e}^{-\left(\frac{x}{\beta }\right)}\right\} \left\{1-\left(1-\gamma \right)\left(1+\frac{x+1}{\beta }\right){e}^{-\left(\frac{x+1}{\beta }\right)}\right\}},$$

and

17 $$\pi \left(\beta |\gamma ,x\right)\propto {\beta }^{{a}_{2}-1}{e}^{-\beta {b}_{2}}\frac{\left\{\left(1+\frac{x}{\beta }\right){e}^{-\left(\frac{x}{\beta }\right)}\right\}- \left\{\left(1+\frac{x+1}{\beta }\right){e}^{-\left(\frac{x+1}{\beta }\right)}\right\} }{\left\{1-\left(1-\gamma \right)\left(1+\frac{x}{\beta }\right){e}^{-\left(\frac{x}{\beta }\right)}\right\} \left\{1-\left(1-\gamma \right)\left(1+\frac{x+1}{\beta }\right){e}^{-\left(\frac{x+1}{\beta }\right)}\right\}}.$$

## Results and discussion

In this section, the results from the Monte Carlo simulation and real-life application are discussed in detail. All numerical calculations performed using R language software.

### Simulation study

1. The following simulation research is carried out to examine the behaviour of Bayesian and maximum likelihood estimates of the DMOLBE distribution. The simulation research is conducted using the below procedures.
2. Generate $N=\mathrm{10,000}$ samples of size $n=50, 100, 150, 200,$ and 300 from DMOLBD.
3. Estimate the parameters $\widehat{\gamma }$ and $\widehat{\beta }$ from each generated sample.
4. Compute the absolute biases (AB) and mean square errors (MSE) using the following equations.

For MLE:

$$Bias\left(\gamma \right)=\frac{1}{N}\sum_{i=1}^{N}\left|\widehat{\gamma }-\gamma \right|\,\&\,Bias\left(\beta \right)=\frac{1}{N}\sum_{i=1}^{N}\left|\widehat{\beta }-\beta \right|,$$

$$MSE\left(\gamma \right)=\frac{1}{N}\sum_{i=1}^{N}{\left(\widehat{\gamma }-\gamma \right)}^{2}\,\&\,MSE\left(\beta \right)=\frac{1}{N}\sum_{i=1}^{N}{\left(\widehat{\beta }-\beta \right)}^{2},$$

For Bayesian

$$Bias\left(\gamma \right)=\frac{1}{N}\sum_{i=1}^{N}\left|\widetilde{\gamma }-\gamma \right|\,\&\,Bias\left(\beta \right)=\frac{1}{N}\sum_{i=1}^{N}\left|\widetilde{\beta }-\beta \right|$$

$$MSE\left(\gamma \right)=\frac{1}{N}\sum_{i=1}^{N}{\left(\widetilde{\gamma }-\gamma \right)}^{2}\,\&\,MSE\left(\beta \right)=\frac{1}{N}\sum_{i=1}^{N}{\left(\widetilde{\beta }-\beta \right)}^{2}$$

The simulation results are reported in Tables 2 and 3. Following conclusions are obtained from the results.

**Table 2:** Simulation results of DMOLBE distribution for different parameter values by MLE estimation.

Parameters	$n$	$AE\left(\widehat{\beta }\right)$	$AE\left(\widehat{\gamma }\right)$	$AB\left(\widehat{\beta }\right)$	$AB\left(\widehat{\gamma }\right)$	$MSE\left(\widehat{\beta }\right)$	$MSE\left(\widehat{\gamma }\right)$
0.5,0.5	50	0.4035	2.1178	-0.0965	1.6178	0.0222	3.6584
	100	0.3958	1.9832	-0.1042	1.4832	0.0155	3.3779
	150	0.3943	1.8475	-0.1057	1.3475	0.0142	1.4960
	200	0.5027	0.5978	0.0027	0.0978	0.0108	0.1274
	300	0.5016	0.5607	0.0016	0.0607	0.0069	0.0603
0.5,1.5	50	0.5194	3.6631	0.0194	2.1631	0.0083	5.6600
	100	0.5471	1.7873	0.0471	0.2873	0.0046	0.9434
	150	0.5408	1.6825	0.0408	0.1825	0.0014	0.5342
	200	0.5234	1.6273	0.0234	0.1273	0.0039	0.3414
	300	0.5119	1.5919	0.0119	0.0919	0.0017	0.2190
1,0.5	50	0.7457	3.4974	-0.2543	2.9974	0.1194	7.0916
	100	0.7381	2.8347	-0.2619	2.3347	0.0888	2.2086
	150	1.0104	0.5652	0.0104	0.0652	0.0426	0.0679
	200	1.0056	0.5513	0.0056	0.0513	0.0316	0.0476
	300	1.0036	0.5336	0.0036	0.0336	0.0202	0.0289
1,1.5	50	0.8720	2.4160	-0.1280	0.9160	0.0492	2.5653
	100	0.9997	1.7286	0.0003	0.2286	0.0267	0.6721
	150	0.9992	1.6427	0.0008	0.1427	0.0173	0.3692
	200	1.0015	1.6025	0.0015	0.1025	0.0131	0.2638
	300	1.0016	1.5650	0.0016	0.0650	0.0089	0.1604
1.5,0.5	50	2.2243	2.0547	0.7243	1.5547	5.2812	4.9220
	100	1.2134	1.7385	-0.2866	1.2385	0.1613	2.3661
	150	1.1985	1.6787	-0.3015	1.1787	0.1385	1.8851
	200	1.2070	1.5835	-0.2930	1.0835	0.1167	1.4551
	300	1.2128	1.5233	-0.2872	1.0233	0.1048	1.2637
1.5,1.5	50	1.3774	2.7501	-0.1226	1.2501	0.1121	2.1164
	100	1.4993	1.7133	0.0007	0.2133	0.0595	0.5899
	150	1.4989	1.6466	0.0011	0.1466	0.0386	0.3683
	200	1.5006	1.6016	0.0006	0.1016	0.0284	0.2470
	300	1.4995	1.5683	0.0005	0.0683	0.0186	0.1541

**Table 3:** Simulation results of DMOLBE distribution for different parameter values by Bayesian estimation.

Parameters	$n$	$AE\left(\widehat{\beta }\right)$	$AE\left(\widehat{\gamma }\right)$	$AB\left(\widehat{\beta }\right)$	$AB\left(\widehat{\gamma }\right)$	$MSE\left(\widehat{\beta }\right)$	$MSE\left(\widehat{\gamma }\right)$
0.5, 0.5	50	0.6380	0.5998	0.1380	0.0998	0.0193	0.0174
	100	0.6277	0.6028	0.1277	0.1028	0.0148	0.0150
	150	0.6050	0.5615	0.1050	0.0615	0.0120	0.0058
	200	0.5244	0.5099	0.0244	0.0099	0.0008	0.0003
	300	0.5161	0.5074	0.0161	0.0074	0.0004	0.0002
0.5, 1.5	50	0.6037	1.5364	0.1037	0.0364	0.0071	0.0093
	100	0.6221	1.5321	0.1221	0.0321	0.0046	0.0076
	150	0.6002	1.5248	0.1002	0.0248	0.0011	0.0030
	200	0.5259	1.5035	0.0259	0.0035	0.0008	0.0003
	300	0.5180	1.5029	0.0180	0.0029	0.0004	0.0001
1, 0.5	50	1.0636	0.6018	0.0636	0.1018	0.0047	0.0168
	100	1.0584	0.6001	0.0584	0.1001	0.0046	0.0143
	150	1.0500	0.5610	0.0500	0.0610	0.0039	0.0056
	200	1.0103	0.5092	0.0103	0.0092	0.0003	0.0003
	300	1.0063	0.5066	0.0063	0.0066	0.0001	0.0001
1, 1.5	50	1.0757	1.5312	0.0757	0.0312	0.0075	0.0086
	100	1.0658	1.5338	0.0658	0.0338	0.0061	0.0068
	150	1.0489	1.5206	0.0489	0.0206	0.0038	0.0030
	200	1.0081	1.5031	0.0081	0.0031	0.0003	0.0003
	300	1.0051	1.5019	0.0051	0.0019	0.0001	0.0001
1.5, 0.5	50	1.5431	0.5819	0.0431	0.0819	0.0053	0.0133
	100	1.5394	0.5828	0.0394	0.0828	0.0042	0.0111
	150	1.5323	0.5452	0.0323	0.0452	0.0029	0.0037
	200	1.5058	0.5060	0.0058	0.0060	0.0003	0.0003
	300	1.5036	0.5048	0.0036	0.0048	0.0001	0.0001
1.5, 1.5	50	1.5430	1.5213	0.0430	0.0213	0.0043	0.0078
	100	1.5392	1.5264	0.0392	0.0264	0.0040	0.0058
	150	1.5257	1.5160	0.0257	0.0160	0.0023	0.0028
	200	1.5043	1.5019	0.0043	0.0019	0.0003	0.0003
	300	1.5022	1.5008	0.0022	0.0008	0.0001	0.0001

## The following points were concluded from the simulation results

1. The estimated bias always decreases and approaches zero when $n\to \infty$ for all combinations of parameters.
2. The estimated MSE decrease with an increase in sample size.
3. Bayesian estimation is better than MLE.

### Applications

This section is based on the advantage of the newly proposed DMOLBE distribution over some commonly used distributions. The performance of the DMOLBED is compared with competitive distributions. The competitive distributions are discrete Burr XII distribution (DBXII), discrete Bilal distribution (DB), discrete Burr–Hatke distribution (DBH), discrete exponentiated Rayleigh distribution (DER), discrete length biased exponential distribution (DLBE), discrete Pareto distribution (DPr), and discrete Poisson distribution (DP). The probability mass functions of these distributions are;

Discrete Burr XII distribution

$$P\left(X=x\right)={\beta }^{\mathrm{ln}\left(1+{x}^{\gamma }\right)} - {\beta }^{\mathrm{ln}\left(1+{\left(1+x\right)}^{\gamma }\right)}$$

Discrete exponentiated Rayleigh distribution

$$P\left(X=x\right)={\beta }^{\mathrm{ln}\left(1+{x}^{\gamma }\right)} - {\beta }^{\mathrm{ln}\left(1+{\left(1+x\right)}^{\gamma }\right)}$$

Discrete Pareto distribution

$$P\left(X=x\right)=\mathrm{exp}\left(-\beta \mathrm{log}\left(1+x\right)\right)-\mathrm{exp}\left(-\beta \mathrm{log}\left(2+x\right)\right)$$

Discrete length Biased exponentiated distribution

$$P\left(X=x\right)=\left(1+\frac{x}{\beta }\right){e}^{-\left(\frac{x}{\beta }\right)}- \left(1+\frac{x+1}{\beta }\right){e}^{-\left(\frac{x+1}{\beta }\right)}$$

Discrete bilal distribution

$$P\left(X=x\right)=2\left({\beta }^{3}-1\right){\beta }^{3x}-3\left({\beta }^{2}-1\right){\beta }^{2x}$$

Discrete Burr–Hatke distribution

$$P\left(X=x\right)=\left(\frac{1}{x+1}-\frac{\beta }{x+2}\right){\beta }^{x}$$

Discrete Poisson distribution

$$P\left(X=x\right)=\frac{{e}^{-\beta }{\beta }^{x}}{x!}$$

The model parameters of considered models are estimated using the maximum likelihood method. The performance of all fitted distributions is compared utilizing some criteria, Akaike information criterion (AIC), Bayesian information criterion (BIC), and Kolmogorov–Smirnov (K–S) test with its corresponding p values. All the computations are carried out in R software.

#### Data Set I (death due to coronavirus in China)

The first data set is the number of deaths due to coronavirus in China from 23 January to 28 March. The data sets used in the paper was collected from 2020 year. The data set is reported in https://www.worldometers.info/coronavirus/country/china/. The data are: 8, 16, 15, 24, 26, 26, 38, 43, 46, 45, 57, 64, 65, 73, 73, 86, 89, 97, 108, 97, 146, 121, 143, 142, 105, 98, 136, 114, 118, 109, 97, 150, 71, 52, 29, 44, 47, 35, 42, 31, 38, 31, 30, 28, 27, 22, 17, 22, 11, 7, 13, 10, 14, 13, 11, 8, 3, 7, 6, 9, 7, 4, 6, 5, 3 and 5. The MLEs with their corresponding standard errors and goodness-of-fit measures are presented in Table 4.

**Table 4:** Parameter estimation and goodness-of-fir measures for first data.

Model	Maximum likelihood estimates (SE)				Model comparison criteria’s			
	$\widehat{\beta }$	SE	$\widehat{\gamma }$	SE	-logL	AIC	BIC	KS (p value)
DMOLBE	39.012	10.339	0.2783	0.1625	327.18	658.35	662.73	0.1285 (0.2255)
DBXII	0.9788	0.0389	6.3999	17.562	374.49	752.99	757.38	0.3607 (0.0000)
DER	34.054	169.93	0.5246	5.2344	347.23	698.45	702.83	0.2932 (0.0000)
DPr	0.2863	0.0352	–	–	379.07	760.14	762.33	0.3816 (0.0000)
DLBE	25.122	2.1866			330.52	663.03	665.22	0.1718 (0.0407)
DB	0.9834	12.114	–	–	330.07	662.14	664.33	0.1655 (0.0538)
DBH	0.9998	0.0019	–	–	461.02	924.04	926.23	0.8119 (0.0000)
Poisson	49.737	0.8681	–	–	1409.8	2821.6	2823.7	0.4975 (0.0000)

**Figure 6:**
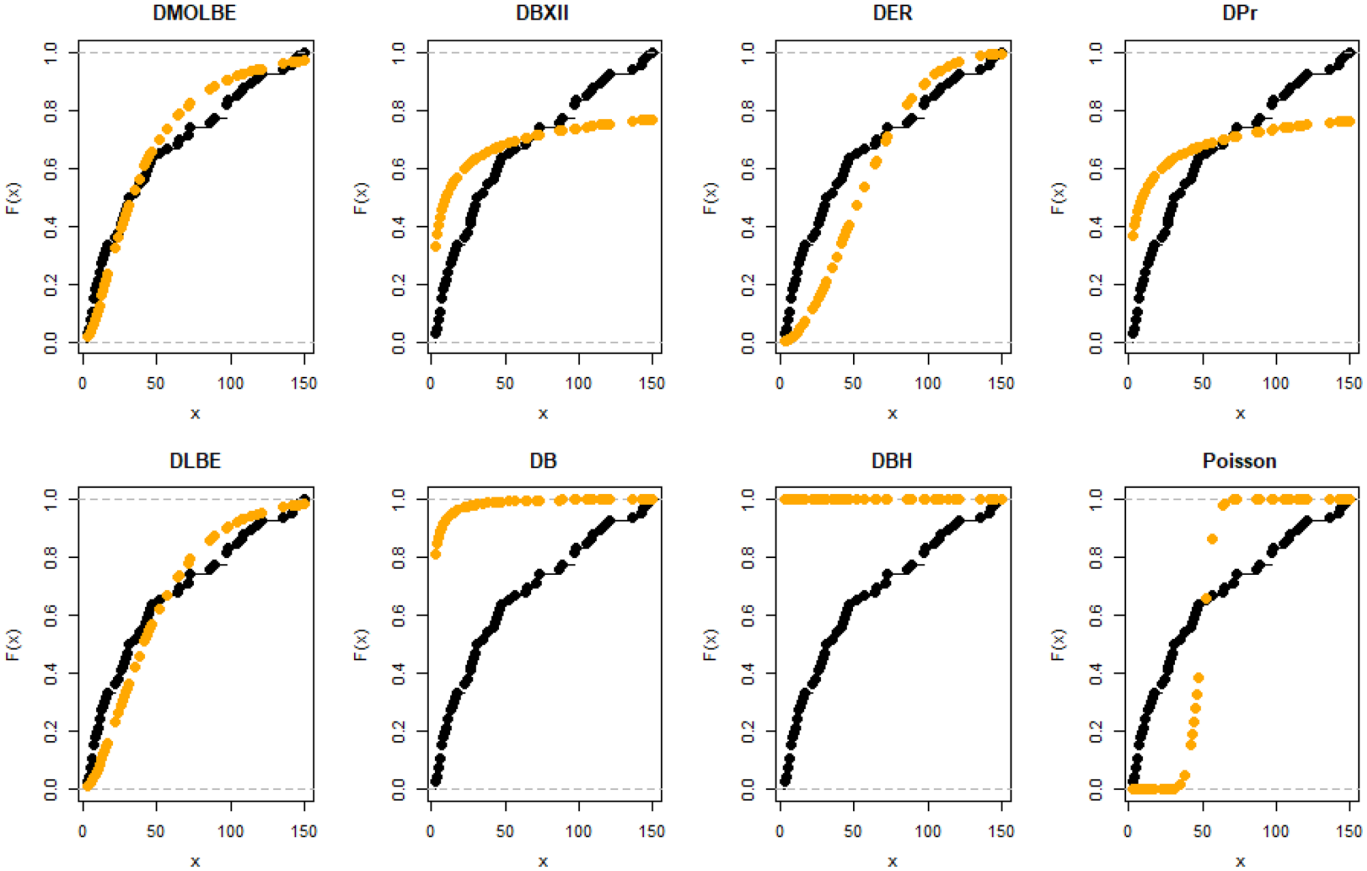
The estimated CDFs for the death due to coronavirus in China.

**Figure 7:**
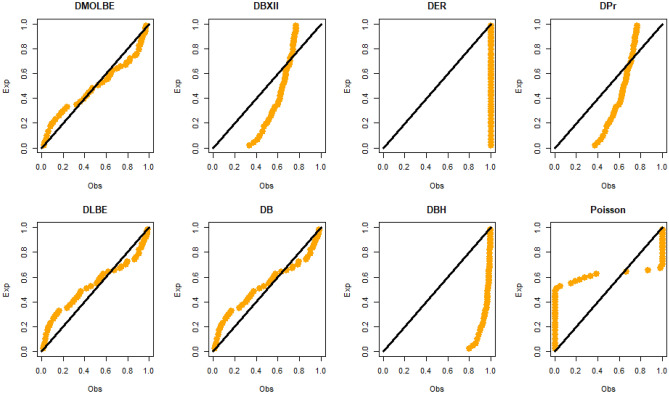
The P–P plots for the death due to coronavirus in China.

**Figure 8:**
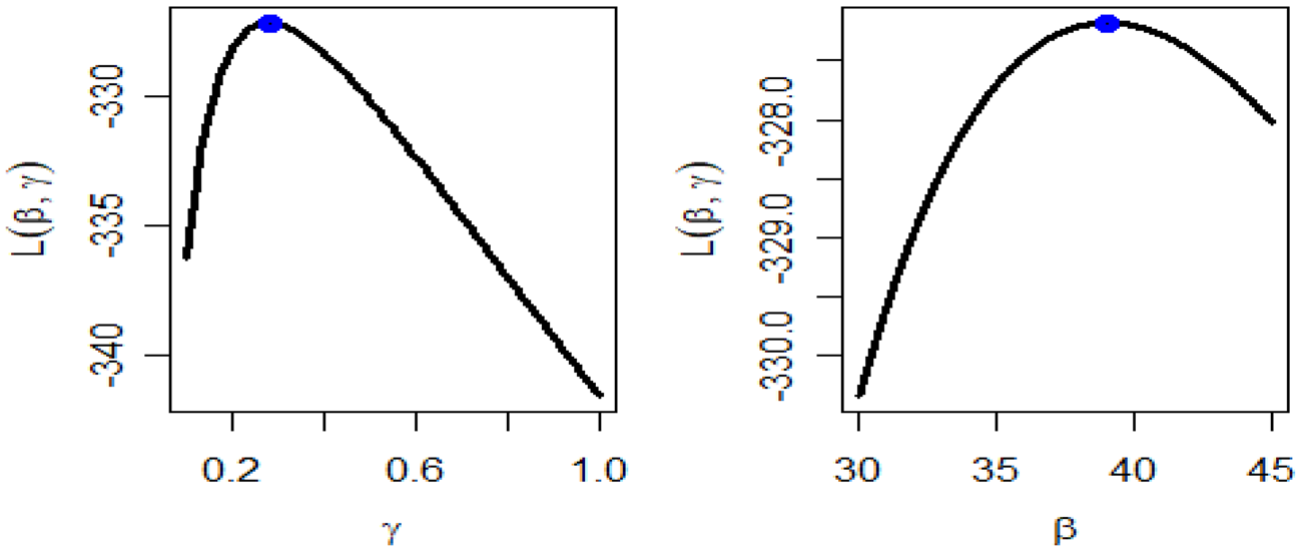
Existence for the log-likelihood for the death due to coronavirus in China.

**Figure 9:**
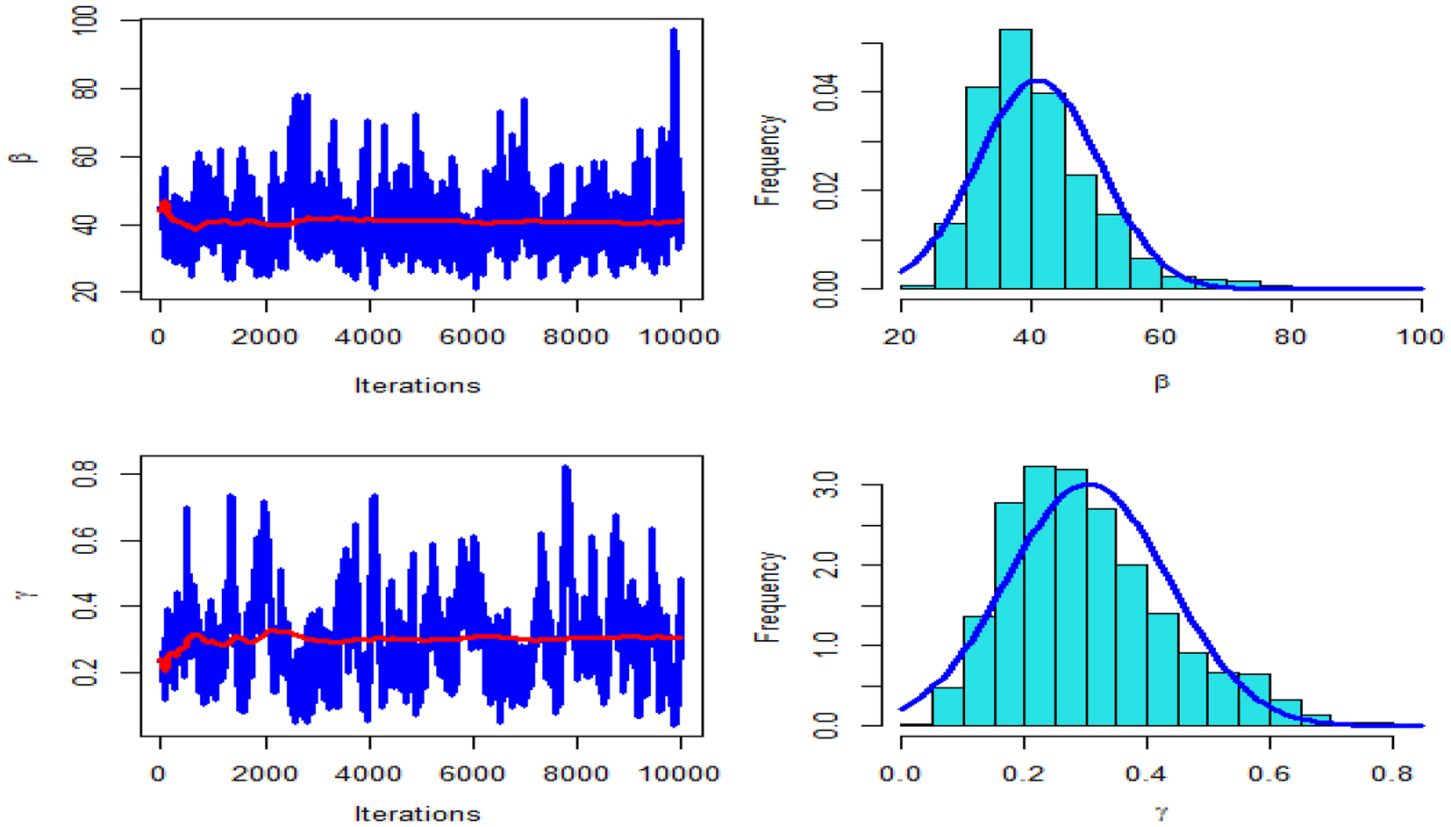
MCMC plots of convergence for parameter estimates of DMOLBED for the death due to coronavirus in China.

Table 4 presents the results for estimated parameters using different models for the first data set which shows that DMOLBE distribution better fits the data set as compared to other competitive models as AIC and BIC are smaller for the proposed model. Table 5 discussed comparing between MLE and Bayesian estimation by SE for the death due to coronavirus in China. By results in Table 5, we conclude that the Bayesian estimation is best estimation method for the death due to coronavirus in China. Figure 6 shows the cdf of different distributions of the first data set and Fig. 7 presents the P–P plots for all the competitive models, both figure supports the results obtained in Table 4. Figure 8 show that estimates of DMOLBED parameters for the death due to coronavirus in China data is existence and has the maximum log-likelihood value. Figure 9 plot MCMC plot results of parameter estimates of DMOLBED for the death due to coronavirus in China data to confirm the estimates have convergence and the posterior has normal distribution as proposed distribution.

**Table 5:** MLE and Bayesian estimation of DMOLBED parameters for the death due to coronavirus in China.

	MLE		Bayesian	
	Estimates	SE	Estimates	SE
$\beta$	39.0118	10.3395	40.9089	9.4192
$\gamma$	0.2783	0.1625	0.3036	0.1324

#### Data Set II (daily death due to coronavirus in Pakistan)

The second data set is the daily deaths due to coronavirus in Pakistan from 18 March to 30 June. The data sets used in the paper was collected from 2020 year. The data is reported in https://www.worldometers.info/coronavirus/country/Pakistan. The data are: 1, 6, 6, 4, 4, 4, 1, 20, 5, 2, 3, 15, 17, 7, 8, 25, 8, 25, 11, 25, 16, 16, 12, 11, 20, 31, 42, 32, 23, 17, 19, 38, 50, 21, 14, 37, 23, 47, 31, 24, 9, 64, 39, 30, 36, 46, 32, 50, 34, 32, 34, 30, 28, 35, 57, 78, 88, 60, 78, 67, 82, 68, 97, 67, 65, 105, 83, 101, 107, 88, 178, 110, 136, 118, 136, 153, 119, 89, 105, 60, 148, 59, 73, 83, 49, 137 and 91.

**Figure 10:**
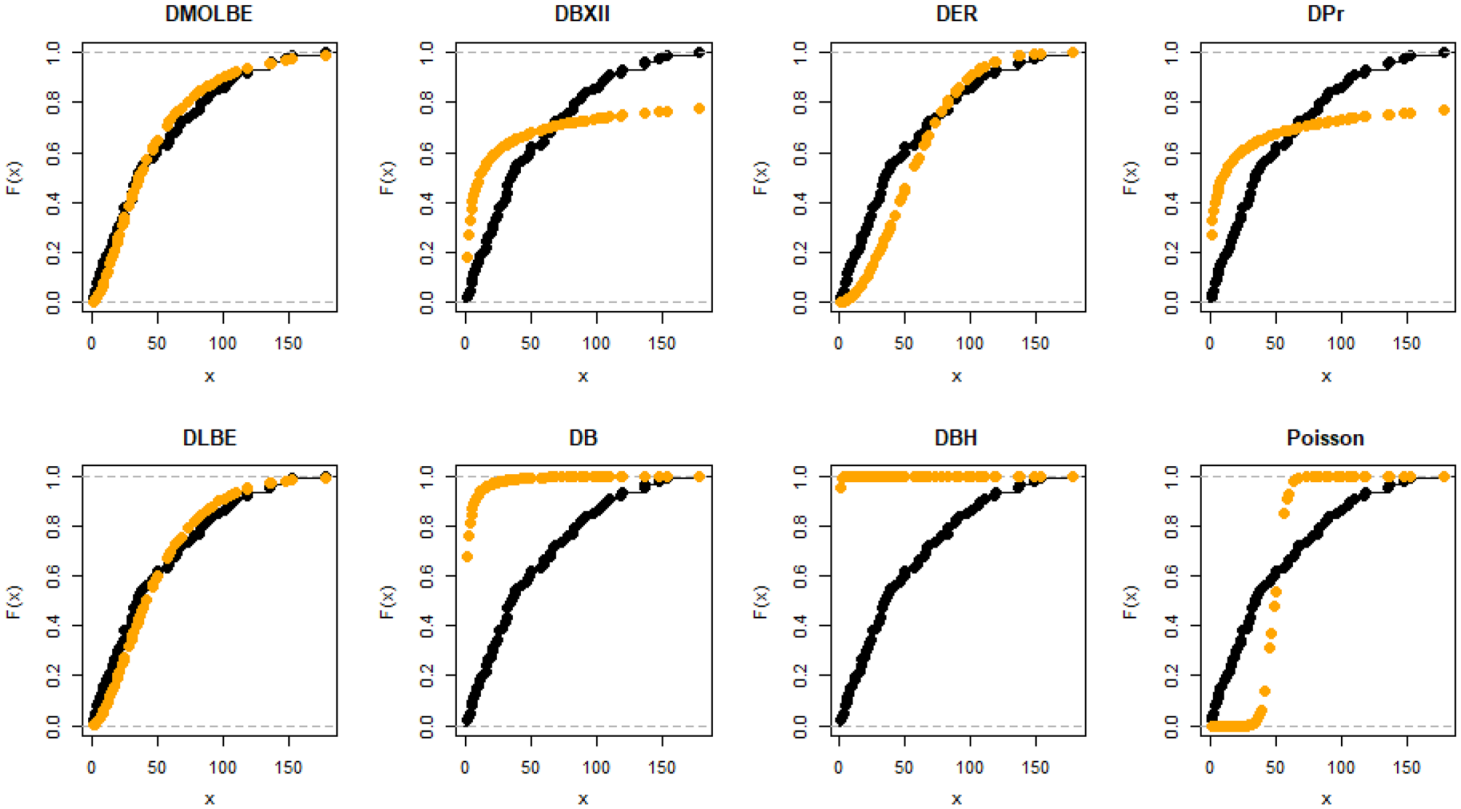
The estimated CDFs for the death due to coronavirus in Pakistan.

**Figure 11:**
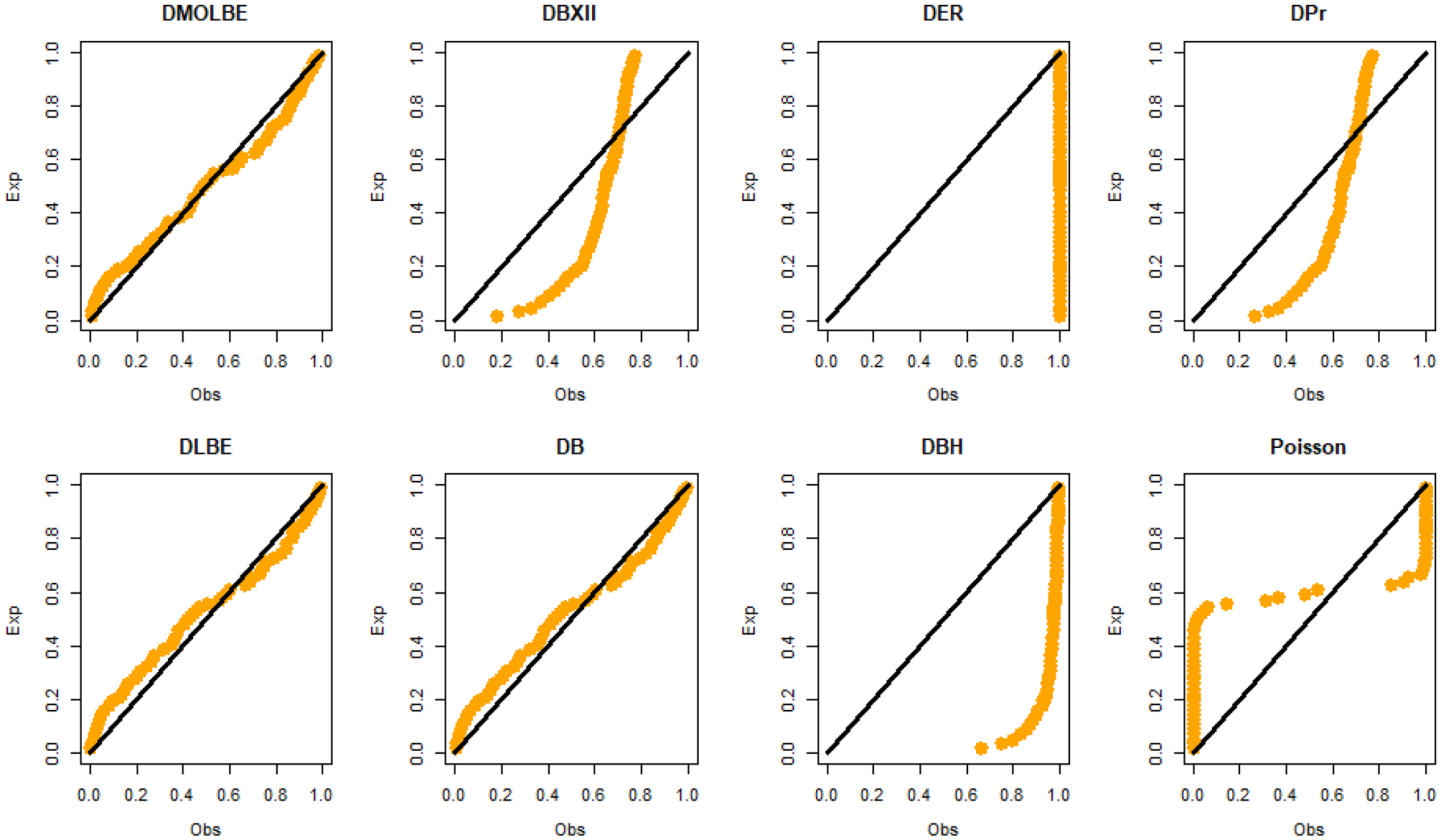
The P–P plots for the death due to coronavirus in Pakistan.

**Figure 12:**
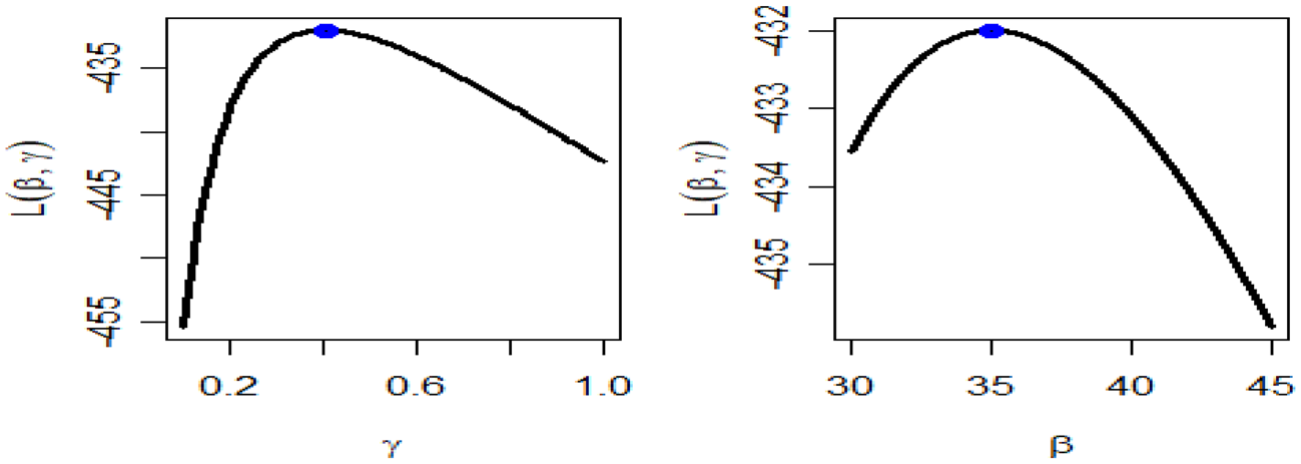
Existence for the log-likelihood of DMOLBED parameters for Coronavirus in Pakistan data.

**Figure 13:**
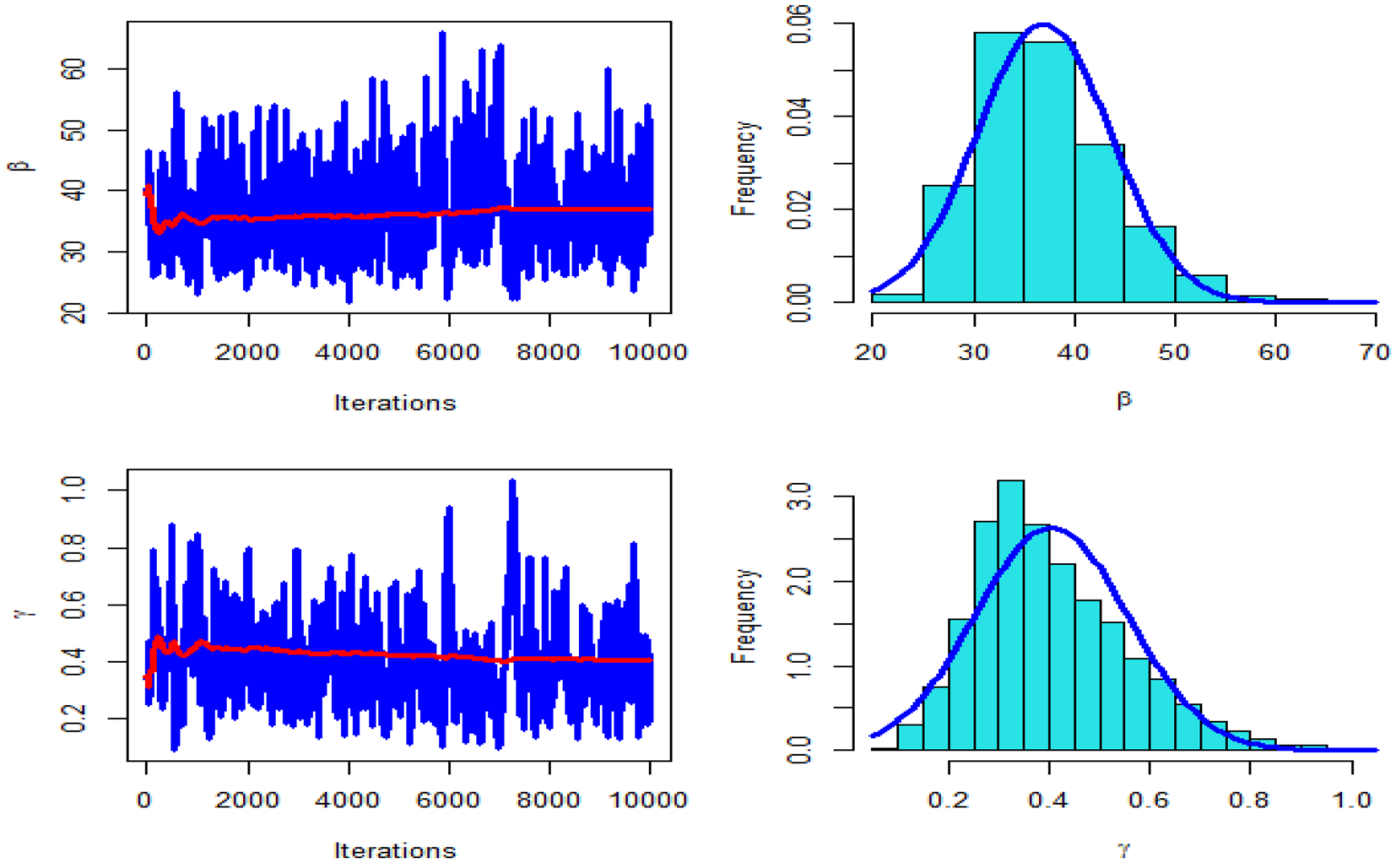
MCMC plots of convergence for parameter estimates of DMOLBED parameters for Coronavirus in Pakistan data.

Table 6 presents the results for estimated parameters using different models of the second data set which shows that DMOLBE distribution better fits the data set as compared to other competitive models as AIC and BIC are smaller for the proposed model. Table 7 discussed comparing between MLE and Bayesian estimation by SE. By results in Table 7, we conclude that the Bayesian estimation is best estimation method. Figure 10 shows the cdf of different distributions of the second data set and Fig. 11 presents the P–P plots for all the competitive models, both figure supports the results obtained in Table 6. Figure 12 show that estimates of DMOLBED parameters for Coronavirus in Pakistan data is existence and has the maximum log-likelihood value. Figure 13 plot MCMC plot results of parameter estimates of DMOLBED for Coronavirus in Pakistan data to confirm the estimates have convergence and the posterior has normal distribution as proposed distribution.

**Table 6:** Parameter estimation and goodness-of-fir measures for second data.

Model	Maximum likelihood estimates (S.E.)				Model comparison criteria’s			
	$\widehat{\beta }$	SE	$\widehat{\gamma }$	SE	-logL	AIC	BIC	KS (p value)
DMOLBE	34.971	7.7459	0.4029	0.2006	431.99	867.99	872.93	0.0922 (0.4499)
DBXII	0.9816	0.0227	15.499	19.249	497.12	998.24	1003.2	0.3500 (0.0000)
DER	33.716	228.83	0.5293	7.1851	452.54	909.09	914.02	0.2473 (0.0000)
DPr	0.2834	0.03038	–	–	503.61	1009.2	1011.6	0.3556 (0.0000)
DLBE	25.279	1.9165	–	–	434.11	870.22	872.68	0.1046 (0.2973)
DB	0.9836	0.1266	–	–	434.69	871.39	877.86	0.0996 (0.3531)
DBH	0.9997	0.0016	–	–	613.80	1229.6	1232.1	0.7876 (0.0000)
DP	50.057	1713.0	–	–	1713.0	3428.1	3430.5	0.4579 (0.0000)

**Table 7:** MLE and Bayesian estimation of DMOLBED parameters for Coronavirus in Pakistan data.

	MLE		Bayesian	
	Estimates	SE	Estimates	SE
Theta	34.9636	7.7266	36.9621	6.6547
Lambda	0.4029	0.2002	0.4042	0.1517

## Conclusion

The DMOLBE distribution, a novel two-parameter discrete probability distribution that may be utilised in place of well-known distributions, is introduced in this study. Its mathematical characteristics are provided in some cases. The maximum likelihood and Bayesian estimation methods are used to estimate the distribution's parameters. The MCMC method is applied by the MH algorithm to produce the Bayesian estimation method. To evaluate the performance of unidentified parameters based on AB and MSE, simulation research is conducted. MLE and Bayesian estimate methods for the performance parameter of the DMOLBE distribution were compared through simulation. We came to the conclusion that the Bayesian estimation approach is superior for estimating DMOLBE distribution parameter. The flexibility of the model is proved by using two real data sets and is compared with different existing models and the proposed model perform better among other models. Further the estimation of the proposed model can be performed using transforms. We will make future work as extension for this study, we will make a regression analysis to predict the future mortality rates in many countries under considerations.

## Future work

Future work in statistical analysis for COVID-19 data holds great potential in advancing our understanding of the pandemic and informing evidence-based decision-making. One key area of focus is the integration of more comprehensive and diverse datasets, including demographic, socioeconomic, and healthcare variables, to explore the multifaceted aspects of COVID-19's impact on different populations. Advanced machine learning techniques can be applied to identify complex relationships and risk factors associated with the spread, severity, and outcomes of the virus. Furthermore, predictive modeling can be enhanced by incorporating real-time data streams and dynamic factors to provide more accurate and timely forecasts, aiding in proactive planning and resource allocation. Longitudinal studies analyzing the long-term effects of the pandemic and assessing the efficacy of interventions over time will provide valuable insights into the sustainability of public health measures. Additionally, ethical considerations and privacy-preserving methodologies should be integrated into future analyses to ensure data security and protect individuals' rights. Overall, future work in statistical analysis for COVID-19 data will continue to play a pivotal role in guiding public health policies, bolstering preparedness for future outbreaks, and ultimately safeguarding global health.

## Data Availability

All data exists in the paper with all its references.

## Abbreviations

MOLBE: Marshall–Olkin Length Biased Exponential
DMOLBE: Discrete Marshall–Olkin Length Biased Exponential
pmf: Probability mass function
cdf: Cumulative distribution function
hrf: Hazard rate function
DI: Dispersion Index
DBXII: Discrete Burr XII distribution
DB: Discrete Bilal distribution
DBHD: Discrete Burr–Hatke distribution
DR: Discrete Rayleigh distribution
Poisson: Poisson distribution
MLE: Maximum likelihood estimation
